# Privileged (Default) Causal Cognition: A Mathematical Analysis

**DOI:** 10.3389/fpsyg.2018.00498

**Published:** 2018-04-10

**Authors:** David Danks

**Affiliations:** Departments of Philosophy & Psychology, Carnegie Mellon University, Pittsburgh, PA, United States

**Keywords:** causal inference, causal reasoning, functional form, linear model, Noisy-OR

## Abstract

Causal cognition is a key part of human learning, reasoning, and decision-making. In particular, people are capable of learning causal relations from data, and then reasoning and planning using those cognitive representations. While there has been significant normative work on the causal structures that ought to be learned from evidence, there has been relatively little on the functional forms that should (normatively) be used or learned for those qualitative causal relations. Moreover, empirical research on causal inference—learning causal relations from observations and interventions—has found support for multiple different functional forms for causal connections. This paper argues that a combination of conceptual and mathematical constraints leads to a privileged (default) functional form for causal relations. This privileged function is shown to provide a theoretical unification of the widely-used noisy-OR/AND models and linear models, thereby showing how they are complementary rather than competing. This unification thus helps to explain the diverse empirical results, as these different functional forms are “merely” special cases of the more general, more privileged function.

## Introduction

Causation forms a core concept and framework for much of human cognition. We perceive causal relations in the world, such as a child *pushing* a block across the floor. We infer causal connections on the basis of (statistical) evidence across multiple experiments, such as overconsumption of caffeine *producing* muscle tremors. We make predictions on the basis of this causal knowledge, such as the prediction from observations of people wearing sweaters that the roads might be icy. We evaluate possible actions and make decisions on the basis of our causal beliefs, such as the conclusion that studying is a better way to achieve a good test score than wishful thinking. Causal cognition is obviously not the entirety of human cognition, but it is equally obviously a fundamental part of our mental lives (Sloman, 2005).

Given the importance of causal cognition, there has unsurprisingly been an enormous amount of research on the nature of human causal representations, and the cognitive processes by which we learn and reason with those representations. This work has been both descriptive—how do people actually understand and reason about the causal structure of the world?—and normative—how should people learn and use causal relations? In fact, causal cognition has arguably been one of the areas of cognitive science that has most benefitted from the interplay between descriptive and normative analyses (Woodward, 2012).

Consider a concrete example that we will employ throughout this paper. There are many different causes of an individual's heart rate at a moment in time, and people can learn and reason about this broad causal structure. Some of these causal factors function somewhat independently of one another; for instance, physical exertion raises one's heart rate in a different way than ingestion of caffeine or other stimulants. Nonetheless, people seem to have the ability to determine the relevant causal relations from observations, experiments, and other kinds of information, and then to reason coherently about the system in the future (Danks, 2014). Their causal knowledge and cognition reflects a relatively integrated structure that suggests that we need to think carefully about the ways in which these complex causal structures should be and are (at least, by default) learned, represented, and used.

Most normative research on causal cognition has focused on three core questions: (1) How ought people represent causal structures? (2) How ought they learn those cognitive representations of causal structure? (3) How ought they reason with those cognitive representations? Answers to these questions have almost exclusively focused on qualitative causal structure—does *C* cause *E*? or, ought *Caffeine*→*Heart Rate* be part of the learned structure?—where a very common answer is that much of our qualitative causal knowledge ought to be, and probably descriptively is, represented as (something like) a causal graphical model (Griffiths and Tenenbaum, 2005; Holyoak and Cheng, 2011; Danks, 2014).

In contrast, normative questions about the *functional form* of causal relations—what is the (mathematical) form of the quantitative causal relationship between *C* and *E*?—have received comparatively little attention. This paper aims to remedy that gap. That is, the present paper examines whether there is a “privileged” functional form that people ought to use as a default when trying to learn quantitative features of various causal relationships. At a high level, the normative argument in this paper starts with a mathematical argument: if a cognitive agent has certain assumptions about causation, then there is a privileged (in a precise formal sense) functional form she ought to use. Moreover, these assumptions are normatively sensible, at least as a default position. Thus, we have normative grounds to use a particular functional form when we do not have other reasons to contradict those assumptions. Of course, this default is defeasible and could be overridden by other knowledge, but it provides the appropriate starting point.

Descriptively, people do appear to be able to use different functional forms, depending on domain knowledge, data, experience with other causal systems, and so forth (Beckers et al., 2005; Lucas and Griffiths, 2010; Lucas et al., 2014). These studies focused, however, on the ways in which prior experience, domain knowledge, and biases might lead people to select one of a small number of possible functional forms. In addition, they were largely focused on causes and effects that can take only a small number of values, often only two (i.e., “on” and “off”; or “present” and “absent”; or “1” and “0”; or some similar pair of values). In the context of our running examples, these theories and experiments would assume that *Heart Rate* can only be “normal” or “elevated.” In contrast, we ask what functional form ought, on normative or rational grounds, be employed as a default when causal factors and effects can take on wider ranges of values.

Although this normative question has received very little attention in cognitive science, we can draw inspiration (and some guidance) from related investigations in machine learning, and in the philosophy and metaphysics of science. Many scientific domains involve models of causal influences that function (at least somewhat) independently of one another. More generally, scientific models and theories frequently divide the world into distinct processes (typically, causal ones) such that the operation of one process has minimal dependence on—in the best case, true independence^1^ from—other processes. Similarly, there are normative reasons to expect human causal cognition to assume (as a default) these kinds of quasi-independent or quasi-modular causal relations. For instance, causal cognition would be computationally intractable if every effect were represented as the entangled product of many intertwined, interacting causes. Section Solving for a Special Case uses ideas and frameworks from machine learning and philosophy of science to make progress on the (normative) cognitive question.

There has also been significant philosophical debate about the metaphysical and epistemological status of what are sometimes called causal “capacities” (Cartwright, 1989, 1999, 2007; Martin, 2008; see also Heil, 2005) or “mechanisms” (in the spirit of Machamer et al., 2000). The basic idea is that capacities are those causal powers that a cause *C* has purely by virtue of being a *C*; causal capacities are “something they [the causes] can be expected to carry with them from situation to situation” (Cartwright, 1989, p. 145). That is, capacities inhere in *C* rather than arising from the particular situation, and so their operation should be relatively unaffected by other processes in the system. A computational literature has developed in parallel that examines privileged functions for these types of quasi-independent *C*s. The core question of this paper—is there a natural, privileged (default) functional framework for human cognitive representations of causal structures?—thus brings together strands from cognitive science, philosophy of science, and machine learning. One might think that there obviously could be *no* such privileged representation, as “mere” quasi-independence seems too weak for this task, but that response turns out to be mistaken. We turn now to examining the question of a normatively privileged (default) functional form for humans to use in causal learning and reasoning.

## Solving for a special case

### Binary variables and the noisy-OR/AND model

Consider the simplest case: all factors—causes *C*_1_, …, *C*_*n*_ and the potential effect *E*—can be represented as binary variables, and the *C*_*i*_s are all either neutral or generative (i.e., increase the probability of *E*). For instance, we might have *Heart Rate* as the target effect, and two distinct potential causes, *Caffeine* and *Exercise*. Recall that a natural default is to assume some degree of (quasi-) independence between the functioning of the various causes. That is, the base causal strength or influence of *C*_*i*_ should be representable without reference to the states of the other variables. In particular, *C*_*i*_'s qualitative impact on *E* should not depend on the state or causal strength of *C*_*j*_, and it should be monotonic in *C*_*i*_. Having said that, *C*_*i*_'s exact quantitative impact in a particular situation might depend partly on the values of *C*_*j*_ and *E* (e.g., because of saturation effects), though again, the “core” causal strength should be independent of those values.

For this simplest case, there is a privileged mathematical function with origins in nineteenth century mathematics. Suppose first that we have a single generative (binary) cause *C*_1_ of the (binary) effect *E*, and so *E* occurs when (and only when) *C*_1_ is present and *C*_1_'s causal influence is “active,” where *w*_1_ is the strength of that capacity (in this context, the probability that it is active). Mathematically, *P*(*E*) = *w*_1_ × δ(*C*_1_), where δ(*X*) = 1 if *X* is present, 0 if *X* is absent. If we have a second generative cause *C*_2_ of *E*, then *E* occurs when (and only when) either *C*_1_ or *C*_2_ generates it, where the “or” is non-exclusive. Mathematically, *P*(*E*) = *w*_1_δ(*C*_1_) + *w*_2_δ(*C*_2_) – *w*_1_δ(*C*_1_)*w*_2_δ(*C*_2_); that is, the probability of *E* is just the sum of the probabilities that it is caused by one or the other cause, minus the probability that both caused it (since that case is “double-counted” in the sum of the first two terms). To return to our running example (and naming variables by their first letters), we have the general equation *P*(*HR* = high | *C, E*) = *w*_*C*_δ(*C*) + *w*_*E*_δ(*E*) − *w*_*C*_δ(*C*)*w*_*E*_δ(*E*). If both *C* and *E* are present, for instance, then *P*(*HR* = high | *C, E*) = *w*_*C*_ + *w*_*E*_ – *w*_*C*_ *w*_*E*_.

More generally, if we have *n* distinct, independent generative causes, then there is a uniquely privileged mathematical function for *P*(*E*) given in Equation (1) below. That is, Equation (1) is the *only* equation for purely generative binary causes with distinct causal capacities (i.e., independent causal influences) that satisfies natural properties (Cozman, 2004) discussed further in section A Privileged, General Mathematical Function.

(1) $$P\left(E|C_{1},\;\ldots ,\;C_{n}\right)=1-\prod_{i\;=\;1}^{n}\left(1-w_{i}\delta \left(C_{i}\right)\right)$$

Equation (1) corresponds mathematically to the so-called “noisy-OR” model (Good, 1961; Kim and Pearl, 1983; Pearl, 1988; Srinivas, 1993; Heckerman and Breese, 1994, 1996; Cheng, 1997; Glymour, 1998; Cozman, 2004). In a noisy-OR model, *E* is a logical OR-function of the different causes, but where each cause has some probabilistic “noise” (understood instrumentally) that can prevent it from bringing about the effect. The probability that *E* occurs is thus the probability that at least one present cause has an active capacity.

Of course, not all causes are generative; we are often interested in causes that *prevent* the effect from occurring. One possibility is that a preventive cause *P* might interfere with the functioning of exactly one specific generative cause *G*. In that case, *P* has the (mathematical) impact of reducing *G*'s causal strength (i.e., the *w*_*G*_ value), and so we can combine *P* and *G* into a single factor with a reduced (relative to the original *w*_*G*_) causal strength. Other times, though, a preventer might impact^2^ *E* in a non-cause-specific way by serving as a (noisy, probabilistic) “switch” that controls whether *any* generative cause can be active at all. In such cases, *E* will occur if and only if (a) at least one generative capacity is active, and (b) none of the preventive causes' capacities are active.

This more complex relationship is captured by the so-called noisy-OR/AND model given in Equation (2). In this equation, the generative causes combine in a noisy-OR function, whose result is then combined with a noisy-AND function for the preventive causes (i.e., the effect occurs only if a generator is active AND *P*_1_ is not active AND … *P*_*m*_ is not active):

(2) $$\begin{array}{l} P\left(E|C_{1},\;\ldots ,\;C_{n},\;P_{1},\;\ldots ,\;P_{m}\right)=\prod_{j=1}^{m}\left(1-w_{j}\delta \left(P_{j}\right)\right)\\ \;\left[1-\prod_{i=1}^{n}\left(1-w_{i}\delta (C_{i}\right)\right] \end{array}$$

This equation provides (arguably) the most natural normative representation, when we have binary variables, of generative and preventive causal capacities that exert independent causal influence (Srinivas, 1993; Heckerman and Breese, 1994, 1996; Lucas, 2005). Equation (2) also reveals the computational simplification provided by thinking in terms of independent causal influences: if we have *n* total generative and preventive causes, then the unrestricted probabilistic model requires 2^*n*^ parameters, but Equation (2) requires only *n* parameters (namely, the *w*-values).

Equation (2) is normatively defensible as the unique privileged function satisfying our natural, default assumptions about quasi-independent binary causes and effects (Cozman, 2004). There is also substantial descriptive evidence that humans often preferentially represent causal systems using functions with the form of Equation (2) (e.g., Cheng, 1997; and the references in Holyoak and Cheng, 2011; Danks, 2014). In cognitive science, Equation (2) is better-known as the “causal power” or “power PC” theory (Cheng, 1997), which was explicitly modeled on Cartwright's (1989) philosophical theory of causal capacities. Although the focus of the present paper is on normatively defensible default or privileged causal functions, it is nonetheless useful confirmation that, at least for this simple case, many people seem to use it in experiments that provide limited domain information. Of course, it is also the case that many people do *not* seem to use this function; we return to this issue in section Psychological (and Other) Implications.

Although Equation (2) is mathematically and conceptually defensible as the privileged (default) function for causal relations, its applicability is highly restricted. There are many cases in which the variables are not binary, but rather have varying magnitudes and impacts. One minimal generalization of the noisy-OR model in Equation (1) is to allow the effect *E* to assume a range of values, typically from zero to positive infinity. This shift to real-valued *E* requires functions that output (*E*), the expectation of *E*, rather than *P*(*E*). Two different generalized functions have traditionally been proposed (Heckerman and Breese, 1996):

$$\begin{array}{l} \text{noisy-MAX model}:\;𝔼\left(E\right)=\max(w_{\text{i}}\delta \left(C_{i}\right))\\ \text{noisy-addition model}:\;𝔼\left(E\right)=\sum {\text{w}}_{\text{i}}\delta \left(C_{i}\right) \end{array}$$

While these are each more general than the noisy-OR model of Equation (1), they are also still highly restricted, as the causes remain binary and there are no preventive causes. Moreover, the fact that there are two different generalizations raises natural questions about which, if either, is the privileged default mathematical function for this case. We provide an answer to that question in section A Privileged, General Mathematical Function, but we must first clarify two key conceptual (though not mathematical) ambiguities produced by the use of binary variables.

### Resolving ambiguities

Mathematically speaking, binary variables are simply those with two possible values. In practice, though, a more specific interpretation is typically intended, particularly when using the noisy-OR/AND model: factors can be “present” vs. “absent” or “on” vs. “off”; capacities can be “active” vs. “inactive.” These interpretations provide a natural value ordering, as shown by the standard practice of mapping “present” to the value of 1 and “absent” to the value of 0^3^. More generally, we typically understand the “absent” or 0 value to be the *lower bound* of the possible values for that variable. At the same time, the 0 value also almost always serves as the *baseline* value: it is the value that *E* would have if nothing influenced it. This second role of the zero value is clear in the mathematics of the noisy-OR/AND model (Equation 2), as *P*(*E* = 0 | all generative causes are absent) = 1. That is, the standard model of (binary) causal capacities assumes that absence is the appropriate “uncaused” state for *E*.

These two different roles for 0—lower bound and baseline value—are conceptually distinct and empirically distinguishable. For example, in most terrestrial environments, the baseline value for *Oxygen in Room* (i.e., the value it has when represented causes are all inactive) is “present,” not “absent.” In the standard noisy-OR/AND model, we can only capture cases in which the lower bound and baseline diverge by using a mathematical trick (namely, a very strong, always-present generative cause). A better solution would be to explicitly allow the lower bound and baseline to diverge. For binary variables, this move does not matter mathematically, as any model with variables whose baseline is 1 can be translated into a model in which all baselines are 0. If any variables are non-binary, however, then the baseline value plays a distinct mathematical role from the lower bound. For example, if *Heart Rate* can range over three values {low, normal, elevated}, then the baseline value is no longer the lower bound.

The multiple roles played by 0 point toward the other important ambiguity (previously mentioned in footnote 2) in the standard noisy-OR/AND model of causal capacities. In general, there are two different ways to prevent *E*, or make *E* less likely. First, the preventer could stop generative causes from exerting their usual influence. These *blockers* serve to keep the effect variable closer to its baseline value, as they (potentially) eliminate causal influences that drive the effect away from baseline. Preventive causes in the noisy-OR/AND model are usually understood in this way. A second way of “preventing” is to move *E* toward its lower bound. These *reducers* are the natural opposite of standard generative causes, as they shift *E* downwards while generators shift *E* upwards. The important distinction here is whether the preventer influences the effect directly (i.e., is a reducer), or indirectly through the elimination of other causal influences (i.e., is a blocker). While these are conceptually distinct types of “prevention,” they are mathematically indistinguishable for binary variables where the baseline and lower bound are the same.

*Heart Rate* provides a ready example of the difference between reducing and blocking. Beta blockers and other anxiety-reducing medications function as blockers, as they prevent (some of) the normal generative causes from having any influence while not suppressing *Heart Rate* below its natural baseline (for that individual). In contrast, most anesthetics are reducers of *Heart Rate*, as they actively slow the heart, potentially even below its natural baseline, depending on exactly which causes are active. If we model *Heart Rate* as simply “low” or “high” (where “low” is the baseline), then these two different types of drugs will appear indistinguishable. In contrast, if we use a three-valued variable {low, normal, elevated}, then the distinction between blockers and reducers becomes clear: the former increase *P*(*HR* = normal), while the latter increase *P*(*HR* = low).

These various distinctions—baseline value vs. lower bound, and blocking vs. reducing—also show that we must clarify what is meant by a “causal strength” *w*_*i*_. The standard interpretation in the noisy-OR/AND model is that *w*_*i*_ expresses the probability that the causal influence or capacity is “active,” in which case it deterministically produces the effect (unless a suitable blocker is also active). If causes are more than binary, though, then this “probability of activation” interpretation neglects the (presumed) importance of the *magnitude* of the cause variable. In this paper, we instead understand *w*_*i*_ (for generators and reducers) to be the expected change in *E*'s value when (a) *C*_*i*_ increases by one unit from its “inactive” state, and (b) every other factor is also inactive. That is, *w*_*i*_ is computed by starting in the state in which no causal factor is active, and then determining the *expected* change in *E* when *C* increases by one unit. Direct measurement of *w*_*i*_ might be a challenge, as it might be difficult or impossible to force all other causal factors to be inactive. Nonetheless, this characterization of *w*_*i*_ is well-defined and conceptually coherent. If all causes and the effect are binary, then the expected change and probability of activation interpretations of *w*_*i*_ are mathematically identical. The expected change interpretation, however, also naturally applies to systems in which some factors can take on more-than-two values.

One potential concern about this expected change interpretation is that, as currently stated, it assumes linearity: causal strength can be captured by measuring the impact on *E* of a one-unit increase in *C*_*i*_ from its baseline (when every other factor is also at its baseline). However, this assumption is relatively innocuous: the default assumption that the *C*s are “modular” causes of *E* implies that we can always independently rescale any non-linear cause *C*_*i*_ (in a non-linear manner) so that *C*_i_^*^ is an appropriately linear cause. Having said that, all subsequent claims still hold—albeit, in a significantly more complicated way, including the inability to use single numbers for causal strengths—even if we assume only that the *C*s are additive causes, not necessarily linear ones.

## A privileged, general mathematical function

We now turn to the overarching topic of this paper: a general, privileged (default) functional form for causal relations. For mathematical tractability, we assume that each variable's possible values can be represented as numbers, though each variable can have its own scale. This assumption is trivial when the variables are binary (i.e., two-valued) and defensible for many non-binary values, but is not always sensible (e.g., there is no privileged way to map the value range {red, green, blue} to numbers). Throughout, we use lower-case letters to denote the value of a variable; for example, *e* is the value of the effect *E*. Without loss of generality, we can assume: *E*'s baseline value is 0; *e* has a lower bound of –*L*; *e* has an upper bound of *U*; and at least one of *L, U* is greater than zero (else *E* is always zero). Note that the baseline can be the same as the lower bound (*L* = 0, *U* > 0); same as the upper bound (*L* > 0; *U* = 0); or a strictly intermediate value (*L, U* > 0).

Three different types of causal capacities must be incorporated into the mathematical framework: generators *G*_*i*_ and reducers *R*_*j*_ that (probabilistically) increase and decrease the value of *E*, respectively; and blockers *B*_*k*_ that (probabilistically) prevent any other causal capacities from influencing *E*. For all three types of causes, we represent (without loss of generality) the “inactive” state of each cause by 0. For generators and reducers, this assumption has the mathematically nice implication that the influence on *E* when only *C* is active is the product of *C*'s magnitude (i.e., its distance from zero) and its causal strength (i.e., the expected change in *E* given that the cause increased by one unit).

Consider the natural, default assumptions for the simplest case in which there are only generators *G*_*i*_ with values *g*_*i*_ and strengths *w*_*i*_. In this situation, *E* can only be pushed upwards from its baseline, and so *e* ∈[0, *U*]. We are interested in the expectation of *E* given the values of the causes, which is given by a function (𝔼) = *f* (**x**), where **x** is the vector of *w*_*i*_*g*_*i*_ products. In a temporary abuse of notation, we will refer to *f* (**x**) as one function, even though **x** could have varying lengths (i.e., numbers of potential causes) for different causal systems. Given our conceptual intuitions about (quasi-)independence, causal capacities, and so forth, I contend that the following four assumptions should hold for any default (mathematical) representation of *f* (**x**):^4^

**Symmetry** (S): *f* (**x**) = *f* (**x**_π_), where **x**_π_ is any permutation of the values of **x**.

**Commutativity** (C): *f* (*f* (**x**, **y**), **z**) = *f* (**x**, *f* (**y**, **z**))

**Associativity** (A): *f* (…, *x, y, z*, …) = *f* (… *f* (*f* (*x, y*), *z*)…) for all *x, y, z*

**Determinism** (D): *f* (**x**) = *e* is a many-one mapping

Assumption S says that the expectation of *E* does not depend on the order in which the causes happen to be listed or represented. Assumptions A and C encode the intuition that, since the causes are (quasi-)independent, the order of incorporation into *f* (**x**) should not matter. Thus, we need not specify *f* (**x**), but only *f* (*x, y*), and so our uses of *f* (**x**) without specifying the length of **x** were, as promised, not actually abuses of notation. Any *f* (**x**) can be computed by repeated application of the two-argument function; for example, *f* (*x, y, z*) = *f* (*f* (*x, y*), *z*). Finally, assumption D encodes the idea that the expectation of *E* should be the same across instances if the causes have the same values, even though the actual value of *E* will almost certainly be different across these cases.

We also have three additional, intuitive default assumptions about *f* (**x**). First, the meaning of “baseline” implies that *E* should be (expected to be) at its baseline value if all causes are inactive (where **0** denotes the vector with 0 for all values):

**No Uncaused Effects**: *f* (**0**) = 0

Second, the characterization of causal strength was that it represents the change in the expectation of *E* when only that cause deviates from its inactive value. For convenience, we use **x**[*y*_*i*_] to denote the vector **x**, but with *y*_*i*_ substituted for *x*_*i*_. Given that, we have:

**Distinct Causal Effect:** *f* (**0**[*y*_*i*_]) = *y*_*i*_

Third, if every generative cause is active and at its maximal value, then the expectation of *E* should presumably be at its maximal (for this model) value. Thus, if **M** denotes the vector in which every cause has its maximal value, then we should have:

**Generative Accumulation**: *f* (**M**) = *U*

Given these assumptions, we now have the following theorem about the uniquely privileged functional form (all proofs provided in the Appendix):

**Theorem 3.1**: Let *f* (**x**) = *e* be a function satisfying SCAD, No Uncaused Effects, Distinct Causal Effect, and Generative Accumulation. If *f* (**x**) is expressible as a finite polynomial^5^, then uniquely $f\left(x,y\right)=x+y-\frac{xy}{U}$

This theorem essentially says: given quite general conditions on how “independent” or modular causes might combine (and an additional, weak condition), there is actually a *unique* way that they must combine. Normatively, there is one function that we ought (mathematically) to use, given these assumptions. In fact, we conjecture that this additional technical condition (of representability by a finite polynomial) can likely be weakened to a “smoothness” condition on *f* (**x**) when it is not at its boundary points.

**Smooth Accumulation**: If *f* (**x**) ≠ *U*, then for ϵ > 0, *f* (**x**[*x*_*i*_+ϵ]) > *f* (**x**).

Roughly, Smooth Accumulation says that a change in any dimension, regardless of the other variable values, should change *f* (**x**) as long as *f* (**x**) is not yet maximal. This condition is satisfied by finite polynomials, but is not satisfied by a function like MAX that depends only on the largest value. We conjecture that the “finite polynomial” condition can be weakened to Smooth Accumulation, though the existence of a proof is currently an open problem.

**Conjecture**: $f\left(x,y\right)=x+y-\frac{xy}{U}$ is the unique function satisfying SCAD, No Uncaused Effects, Distinct Causal Effect, Generative Accumulation, and Smooth Accumulation.

Given the high likelihood that either the conjecture is true, or else the “finite polynomial” condition in Theorem 3.1 can be dropped, then we are justified in concluding that this particular *f* (*x, y*) is the privileged causal function for two, solely generative causes. We can then immediately derive the similarly privileged function for multiple generative causes, as *f* (**x**) is simply the result of repeatedly applying *f* (*x, y*), adding one more cause each time. Since *f* (**x**) provides the expectation of *E*, we can usefully write this privileged generalized function as:^6^

$$𝔼\left(E\right)=U\left[1-\prod_{i=1}^{g}\frac{U-w_{i}g_{i}}{U}\right]$$

Interestingly, this function is directly connected with the noisy-OR model, as it can be understood as: (i) “normalize” *E* and the causal strengths to the [0, 1] interval; (ii) use the noisy-OR model; and then (iii) transform the result back to the [0, *U*] interval. Of course, this function interprets the noisy-OR equations as providing the expectation of *E*, rather than its (conditional) probability.

We can use this connection with the noisy-OR model to generalize the privileged function to include reducing causes, naturally understood as “negative generators.” More specifically, we treat a set of reducers *R*_*j*_ with values *r*_*j*_ as generative causes that have *negative* impact on the expectation, though their “normalization” is relative to *L* rather than *U*. The resulting expectation of *E* is simply the difference between these (normalized and combined) influences:

$$𝔼\left(E\right)=U\left[1-\prod_{i=1}^{g}\frac{U-w_{i}g_{i}}{U}\right]-L\left[1-\prod_{j=1}^{r}\frac{L-w_{j}r_{j}}{L}\right]$$

Finally, blockers *B*_*k*_ with values *b*_*k*_ fill the role of preventers in the noisy-OR/AND model of Equation (2): the (probabilistic) activation of their causal capacities prevents the expression of any other causal capacities, and so they act as a probabilistic “switch” on the previous equation. The causal strengths of the blocking capacities are thus best understood as “increase (per unit change in the blocker) in probability of complete blocking when all other blockers are inactive”^7^. The resulting full mathematical equation is:

(3) $$\begin{array}{l} 𝔼\left(E\right)=\prod_{k=1}^{b}\left(1-w_{k}b_{k}\right)[U\left[1-\prod_{i=1}^{g}\frac{U-w_{i}g_{i}}{U}\right]\\ \;-L\left[1-\prod_{j=1}^{r}\frac{L-w_{j}r_{j}}{L}\right]] \end{array}$$

We contend that Equation (3) is the privileged, default functional form for causal relationships, as all of the relevant assumptions, whether mathematical or conceptual, naturally apply for cognitive agents such as humans. Moreover, many of the functional forms that have previously been proposed as defaults for causal relationships emerge as special cases of Equation (3), even for cases such as *U* = +∞ that seemingly imply infinities throughout Equation (3). In particular, we have the following theorems:

**Theorem 3.2**: If *L* = 0, *U* = 1, and *C*_*i*_ ∈ {0, 1}, then Equation (3) is the noisy-OR/AND model.

**Theorem 3.3**: If *L* = –∞, *U* = +∞, *C*_*i*_ ∈ [–∞, +∞], and there are no blockers, then Equation (3) is $𝔼\left(E\right)=\overset{g}{\underset{i=1}{\sum }}w_{i}g_{i}-\overset{r}{\underset{j=1}{\sum }}w_{j}r_{j}$.

**Corollary 3.4**: If *L* = –∞, *U* = +∞, *C*_*i*_ ∈ {0,1}, and there are only generators, then Equation (3) is the noisy-addition model.

**Theorem 3.5**: If *L* = –∞, *U* = +∞, and *C*_*i*_ ∈ [–∞, +∞], then Equation (3) is $𝔼\left(E\right)=\prod_{k=1}^{b}\left(1-w_{k}b_{k}\right)\left[\overset{g}{\underset{i=1}{\sum }}w_{i}g_{i}-\overset{r}{\underset{j=1}{\sum }}w_{j}r_{j}\right]$

Theorem 3.3 is particularly interesting, as it provides natural conditions in which (the expectation of) *E* is a linear function of the causal capacities. Models in which the expectation of *E* is a linear function of its causes have been widely proposed in the causal cognition literature, either directly in the form of the Δ*P* model (e.g., Cheng and Novick, 1992; Shanks, 1995), or indirectly through the use of associationist models such as (Rescorla and Wagner, 1972; see also generalizations such as Van Hamme and Wasserman, 1994) that equilibrate at the Δ*P* values (Danks, 2003). In all of these models, causes combine linearly, rather than the sub-linear combination that occurs in the noisy-OR/AND model. Theorems 3.2 and 3.3 thus show that the two dominant functional forms proposed in the human causal cognition literature emerge as special cases of Equation (3), depending on the values of the variables^8^.

These theorems are also relevant for causal inference—prediction and reasoning given particular known or inferred causal structures. For example, given the observation that someone has been exercising, I can infer that their heart rate is probably elevated. Causal inference is well-studied for the special cases of causal power and linear causal systems, and that understanding applies straightforwardly to Equation (3) in the relevant special case conditions. Many of the standard phenomena of causal inference (e.g., explaining away^9^) straightforwardly arise even outside of the special case conditions, but the precise inferences that are justified for the more generalized version of Equation (3) are an open question.

## Psychological (and other) implications

Equation (3) provides a measure of unification to the noisy-OR/AND models and linear models: despite their substantial mathematical differences, both are special cases of the more general, privileged functional form. That is, these results suggest that noisy-OR/AND models and linear models actually share a conceptual and mathematical basis; different models arise simply based on whether the variables are binary or continuous/real-valued. This observation suggests that people in causal learning experiments might (descriptively) systematically shift between the noisy-OR/AND functional form and the linear functional form, depending on the variable value ranges. Unfortunately, cover stories for those experiments rarely explicitly provide value ranges, and so it is difficult to determine from current data whether variation in assumed or inferred variable value ranges could explain the diversity of empirical findings.

As one early-but-illustrative example, Lober and Shanks (2000) found a mixture of functions within their experimental participants: many seemed to assume the noisy-OR/AND (or power PC) model, but many others assumed a linear (or Δ*P*) model. Their experimental cover story asked participants to determine the causal impact of various chemical exposures on DNA mutations. From the experimenters' point-of-view, the variables were all binary: animals were exposed or not; mutation occurred or not. However, one might naturally think that some participants interpreted the variables differently: exposure can come in varying degrees, and mutations can have varying impacts or severity. The framework presented in this paper predicts that people normatively ought to change the functional form that they assume depending on how they interpret the variables. If they adopted the experimenters' framing, then they should use the noisy-OR/AND model. If they believed that the factors could vary more smoothly, then they should use the linear model (or something more like the linear model, since *U* might not be +∞). And although people frequently deviate behaviorally from normative prescriptions, these normative guidelines provide reason to suspect that people might think about default causal functions in these ways (since this is the privileged default function).

Varying beliefs about variable value ranges are not the only possible explanation for diversity in functional form. For example, people might instead simply use one or another functional form out of habit, or because of distinctive past experiences. Alternately, the probe question used to obtain causal judgments is also known to influence the responses. In this case, though, we need to be careful to distinguish between two different possibilities. First, different probe questions might actually be eliciting judgments of different values, perhaps parameters in Equation (3) but perhaps not. For example, the different probe questions in Collins and Shanks (2006) arguably elicit judgments of different quantities, all of which appear as (or can be computed from) parameters in Equation (3). A second possibility is that different probe questions elicit judgments of the same parameter, but bias those judgments in specific ways. However, there does not currently seem to be an uncontroversial case demonstrating such an impact of probe question. More generally, though, I contend that varying value range beliefs are plausibly one explanation for diversity in parametric form, but I am not thereby committed to these being the *only* plausible explanation.

Anecdotally, this type of diversity in inferred variable value ranges would explain other cases of potentially puzzling empirical data beyond the Lober and Shanks (2000) experiments. Further targeted experimentation would be required to determine the variable value ranges that people infer from the cover stories. One route would be experiments in which participants observe identical data, but receive different cover stories that frame the variables as either binary or continuous. A different route would be to show logically almost-identical data, but with slightly different values that cue participants to different potential ranges^10^. For example, the sequence < 0, 0, 1, 0, …> suggests a binary variable; < 0.01, −0.02, 1.05, 0.03, …> gives essentially the same information but suggests a continuous variable with narrow value range; and < 0.03, 0.07, 17.32, −0.01, …> suggests a continuous variable with much wider range (but still with only two functionally-distinct values). The present framework predicts that there should be corresponding differences in the assumed functional forms for causal inference as either the cover story or the numeric ranges shift (cf. Beckers et al., 2005). A different route would be to look for systematic variation in causal inference depending on variable ranges since, as noted earlier, the theory expressed here implies that causal reasoning should also use Equation (3).

The privileged Equation (3) also connects with theories of human causal learning, beyond simply the Δ*P* and causal power theories (and their dynamical variants). For example, various Bayesian theories (or theories based on probabilistic inference over causal models) have been proposed for human causal learning (e.g., Steyvers et al., 2003; Griffiths and Tenenbaum, 2005; Lu et al., 2008; and many papers derived from these theories). These (quasi-)Bayesian theories all require that we either specify a functional form for the causal models, or else put a probability distribution over possible functional forms (as in Lucas and Griffiths, 2010). In either case, though, the linear or noisy-OR/AND models are the dominant ones that are used. A natural question is whether a causal (structure) learning theory based on Equation (3) as the functional form would yield different descriptive accuracy.

Outside of causal cognition, there is a long history of psychological research on function approximation that has shown that people find linear functions easier to learn (e.g., DeLosh et al., 1997; McDaniel and Busemeyer, 2005; and references therein), and even have a significant bias in favor of understanding the world in terms of linear functions (Kalish et al., 2007). These results are sometimes thought to be in conflict with the many causal learning experiments showing noisy-OR/AND models to be preferred. In particular, one might *a priori* expect that causal learning and function learning should default to similar functions, as causal inference is arguably a special case of function learning. It would presumably be odd if the two types of learning had completely different defaults. Equation (3) provides a measure of theoretical unification for these disparate psychological results: noisy-OR/AND models and linear models are not theoretical competitors, but rather different special cases of the same general, privileged equation for (quasi-)independent causes or factors. In practice, function learning experiments always use continuous spaces, so the preference for linear functions is expected (given those variable value ranges). More generally, we ought not frame the issue as “noisy-OR/AND models vs. linear models,” since each is the natural representation *for a particular domain of variable values*.

The shared basis in Equation (3) for noisy-OR/AND models and linear models also helps to explain a potentially surprising observation: many mathematical results that hold for linear models also hold for noisy-OR/AND models, and vice versa. For example, the conditions for model parameter identifiability are essentially the same for noisy-OR/AND models (Hyttinen et al., 2011) and linear models (Hyttinen et al., 2012). Similarly, we find basically the same conditions and statistical tests for discovering an unobserved common cause of multiple observed effects given either a noisy-OR/AND model (Pearl, 1988; Danks and Glymour, 2001) or a linear model (Spirtes et al., 2000). The overlap in the models' mathematical properties has been anecdotally noted, but there has not been a clear explanation for why there would be such overlap. The existence of many shared properties is, however, much less surprising given that both models are special cases of a single, more general equation (though their properties are not identical, since the different variable value ranges do sometimes matter). Equation (3) thus also points toward a plausible mathematical framework for the widespread philosophical notion of causal capacity or causal mechanism (Cartwright, 1989, 1999, 2007; Machamer et al., 2000; Heil, 2005; Martin, 2008). Of course, Equation (3) only provides a default or starting point; nonetheless, it can potentially bring additional precision and clarity to philosophical debates about the “natural” metaphysical structure and form of causal relationships.

## Conclusions

Causal learning, knowledge, inference, and reasoning are crucial capabilities for successful action and navigation in the world. At the same time, we frequently lack detailed domain knowledge to determine, in a particular setting, the exact ways that causes might combine to produce an effect. We thus need “defaults” in terms of the functional forms that we use for causal learning and reasoning. Observations, instruction, or other reasoning might override these defaults, but they enable us simply to begin causal learning in the first place. There is a rich cognitive science literature attempting to determine how people think about causal relations, whether before or after they observe evidence. There has, however, been relatively little investigation of the normative question of what functional form they *ought* to assume.

This paper builds on cognitive science and machine learning results for the simple case of all binary causes and effects to develop a generalized framework that applies for arbitrary variable value ranges. The resulting equation provides a privileged default functional form for causal inference when we expect the causal system to be composed of a set of (quasi-)independent causal mechanisms or capacities. This generalized framework provides further conceptual clarification about causal capacities, as it reveals distinctions (e.g., between the lower bound and the baseline value) that have previously been relatively little-explored in the psychological and machine learning literatures. More importantly, this “master equation” provides a natural way to unify disparate equations—in particular, the noisy-OR/AND models and linear models—that have previously been viewed in cognitive science as competing theories, and in machine learning as relatively independent of one another. The widespread use, value, and connections between such models is eminently explainable when we understand them as deriving from the same fundamental framework and equation. This privileged framework thus provides a precise, formal representation that can significantly constrain our normative models of causal capacities, and thereby people's default representations of them.

## Author contributions

DD performed all research and writing for this article.

### Conflict of interest statement

The author declares that the research was conducted in the absence of any commercial or financial relationships that could be construed as a potential conflict of interest.

## Footnotes

^1^This independence should not be confused with either (a) statistical independencies that can be used to (sometimes) infer causal structures from data (Spirtes et al., 2000); or (b) modularity of causal connections that can be separately intervened upon (Hausman and Woodward, 1999, 2004; Cartwright, 2002).

^2^There are two different ways in which a preventer could “impact” *E*, but they collapse together for binary variables. We will disambiguate them in section Resolving Ambiguities.

^3^This mapping could be reversed without any change in substantive content, though “lower bound” and “upper bound” would need to be swapped in what follows.

^4^These assumptions are jointly similar to those in Cozman (2004), but they apply to non-binary variables, and are separated into distinct claims in a somewhat different way.

^5^This antecedent independently implies Determinism, and so we could remove D from the theorem. However, we retain it to preserve the parallel structure of the subsequent Conjecture, since Smooth Accumulation does not imply D.

^6^Theorem 3.3 below shows that this equation is well-behaved even when *U* = +∞.

^7^Note that this interpretation implies that always *w*_*k*_*b*_*k*_ ≤ 1, since their product is the probability of complete blocking if *B*_*k*_ is the only active blocker.

^8^An interesting open question is whether these theorems about 𝔼(*E*) could be generalized to *P*(*E*), the actual probability distribution of *E*. As a promising first step, we can prove: if there is one generative cause and the initial *P*(*E*) is uniform over [–*L, U*], then the “update” equation $P_{new}\left(E=e|G=g\right)=P_{old}\left(E=U\frac{e-w_{g}g}{U-w_{g}g}\right)$ satisfies all of the relevant mathematical and conceptual desiderata. It is unknown whether other results of this type can be obtained.

^9^For example, if I see someone has an elevated heart rate, then that increases the probability that they have been exercising. However, if I subsequently learn that they have had many cups of coffee, then the probability of exercising drops to (almost) baseline, since the known caffeine ingestion “explains away” the elevated heart rate.

^10^Thanks to an anonymous reviewer for suggesting this way to test the relevance of variable type and value ranges.

## Appendix of proofs

This appendix provides the proofs of all theorems (with the theorems included for completeness).

**Theorem 3.1**: Let *f* (**x**)=*e* be a function satisfying SCAD, No Uncaused Effects, Distinct Causal Effect, and Generative Accumulation. If *f* (**x**) is expressible as a finite polynomial, then uniquely $f\left(x,y\right)=x+y-\frac{xy}{U}$.

**Proof of Theorem 3.1**: Any symmetric polynomial in *x, y* is uniquely expressible as a polynomial with (*x*+*y*) and (*xy*) as the base elements: $f\left(x,y\right)=\underset{i\geq 0}{\sum }\alpha_{i}{\left(x+y\right)}^{i}+\underset{j>0}{\sum }\beta_{j}{\left(xy\right)}^{j}$. Since *f* () is finite, then *i, j* < ∞. Let *n* be the largest degree of *x* and *y* in the *f* (*x, y*) polynomial (which is the same for both, by Symmetry). Commutativity implies that *f* (*f* (*x, y*), *z*) = *f* (*f* (*x, z*), *y*). The left-hand side of this equality involves two applications of *f* () to *y*, so its degree will be *n*^2^; the right-hand side involves only one application of *f* () to *y*, so its degree will be *n*. Therefore, the equality can only hold if *n* = 0 or *n* = 1. Thus, necessarily we have: *f*(*x, y*) = *C* + α(*x*+*y*) + β(*xy*), for some constant *C* (and where possibly *C*, α, or β equal 0). By No Uncaused Effects, *f* (0,0) = 0 and so *C* = 0. By Distinct Causal Effect, *f* (*x*, 0) = *x* and so α = 1. Without loss of generality, we can assume that all factors have the same maximal value *U*. Then by Generative Accumulation, *f* (*U, U*) = *U* and so $\beta =\frac{-1}{U}$. Hence, $f\left(x,y\right)=x+y-\frac{xy}{U}$.

**Theorem 3.2**: If *L* = 0, *U* = 1, and *C*_i_ ∈ {0, 1}, then Equation (3) is the noisy-OR/AND model.

**Proof of Theorem 3.2**: Since *L* is equal to the baseline, there are no “reducing” causal capacities: for any putative reducer *R*, the expected change in *E* from a unit change in *R* (when all other causes are absent) is always zero, and so *w*_R_ is always zero. Since the causal factors are restricted to {0, 1}, the *b*_k_ and *g*_i_ variable values can be replaced with delta functions. The resulting equation (when we substitute in *U* and *L*) is the noisy-OR/AND model.

**Theorem 3.3**: If *L* = −∞, *U* = +∞, *C*_i_ ∈ [−∞, +∞], and there are no blockers, then Equation (3) is $𝔼\left(E\right)=\overset{g}{\underset{i=1}{\sum }}w_{i}g_{i}-\overset{r}{\underset{j=1}{\sum }}w_{j}r_{j}$.

**Proof of Theorem 3.3**: Consider only the generators in Equation (3). Algebraic transformation yields $U1-(\left[1-\overset{g}{\underset{i=1}{\sum }}\frac{w_{i}g_{i}}{U}+\Gamma \right)]=\overset{g}{\underset{i=1}{\sum }}w_{i}g_{i}+U\Gamma$, where Γ is the remainder of the product expansion. Every term in Γ has at least *U*^2^ in the denominator, and so as *U* → +∞, *U*Γ → 0. The same reasoning yields the corresponding summation for reducers.

**Corollary 3.4**: If *L* = −∞, *U* = +∞, *C*_i_ ∈ {0,1}, and there are only generators, then Equation (3) is the noisy-addition model.

**Proof of Corollary 3.4**: The restriction of the *C*_i_ values implies we can use δ(*G*_i_) rather than *g*_i_. Direct substitution into the equation in Theorem 3.3 yields the noisy-addition model.

**Theorem 3.5**: If *L* = −∞, *U* = +∞, and *C*_i_ ∈ [−∞, +∞], then Equation (3) is $𝔼\left(E\right)=\prod_{k=1}^{b}\left(1-w_{k}b_{k}\right)\left[\overset{g}{\underset{i=1}{\sum }}w_{i}g_{i}-\overset{r}{\underset{j=1}{\sum }}w_{j}r_{j}\right]$.

**Proof of Theorem 3.5**: It is straightforward to incorporate blockers into Theorem 3.3, as the initial product term in Equation (3) will simply act to globally attenuate the linear impact (on the expectation of *E*) of the generators and reducers.

## References

[B1] Beckers T. De Houwer J. Pineño O. Miller R. R. (2005). Outcome additivity and outcome maximality influence cue competition in human causal learning. J. Exp. Psychol. 31, 238–249. 10.1037/0278-7393.31.2.238 15755242

[B2] Cartwright N. (1989). Nature's Capacities and their Measurement. Oxford: Oxford University Press.

[B3] Cartwright N. (1999). The Dappled World: A Study of the Boundaries of Science. Cambridge: Cambridge University Press.

[B4] Cartwright N. (2002). Against modularity, the causal Markov condition, and any link between the two: comments on Hausman and Woodward. Br. J. Philos. Sci. 53, 411–453. 10.1093/bjps/53.3.411

[B5] Cartwright N. (2007). Hunting Causes and Using them: Approaches in Philosophy and Economics. Cambridge: Cambridge University Press.

[B6] Cheng P. W. (1997). From covariation to causation: a causal power theory. Psychol. Rev. 104, 367–405. 10.1037/0033-295X.104.2.367

[B7] Cheng P. W. Novick L. R. (1992). Covariation in natural causal induction. Psychol. Rev. 99, 365–382. 10.1037/0033-295X.99.2.365 1594730

[B8] Collins D. J. Shanks D. R. (2006). Conformity to the power PC theory of causal induction depends on the type of probe question. Q. J. Exp. Psychol. 59, 225–232. 10.1080/17470210500370457 16618631

[B9] Cozman F. G. (2004). Axiomatizing noisy-OR, in Proceedings of the 16th European Conference on Artificial Intelligence (Amsterdam: IOS Press).

[B10] Danks D. (2003). Equilibria of the Rescorla-Wagner model. J. Math. Psychol. 47, 109–121. 10.1016/S0022-2496(02)00016-0

[B11] Danks D. (2014). Unifying the Mind: Cognitive Representations as Graphical Models. Cambridge, MA: The MIT Press.

[B12] Danks D. Glymour C. (2001). Linearity properties of Bayes nets with binary variables, in Proceedings of the 17th Annual Conference on Uncertainty in Artificial Intelligence, eds Breese J. Koller D. (San Francisco, CA: Morgan Kaufmann), 98–104.

[B13] DeLosh E. L. Busemeyer J. R. McDaniel M. A. (1997). Extrapolation: the sine qua non for abstraction in function learning. J. Exp. Psychol. Learn. Mem. Cogn. 23, 968–986. 10.1037/0278-7393.23.4.968 9231439

[B14] Glymour C. (1998). Learning causes: psychological explanations of causal explanation. Minds Mach. 8, 39–60. 10.1023/A:1008234330618

[B15] Good I. J. (1961). A causal calculus (I). Br. J. Philos. Sci. 11, 305–318. 10.1093/bjps/XI.44.305

[B16] Griffiths T. L. Tenenbaum J. B. (2005). Structure and strength in causal induction. Cogn. Psychol. 51, 334–384. 10.1016/j.cogpsych.2005.05.004 16168981

[B17] Hausman D. M. Woodward J. (1999). Independence, invariance and the causal Markov condition. Br. J. Philos. Sci. 50, 521–583. 10.1093/bjps/50.4.521

[B18] Hausman D. M. Woodward J. (2004). Modularity and the causal Markov assumption: a restatement. Br. J. Philos. Sci. 55, 147–161. 10.1093/bjps/55.1.147

[B19] Heckerman D. Breese J. S. (1994). A new look at causal independence, in Proceedings of the 10th Annual Conference on Uncertainty in Artificial Intelligence (San Francisco, CA: Morgan Kaufmann), 286–292.

[B20] Heckerman D. Breese J. S. (1996). Causal independence for probability assessment and inference using Bayesian networks. IEEE Trans. Syst. Man Cybern. A Syst. Humans 26, 826–831. 10.1109/3468.541341

[B21] Heil J. (2005). From an Ontological Point of View. NewYork, NY: Oxford University Press.

[B22] Holyoak K. J. Cheng P. W. (2011). Causal learning and inference as a rational process: the new synthesis. Annu. Rev. Psychol. 62, 135–163. 10.1146/annurev.psych.121208.131634 21126179

[B23] Hyttinen A. Eberhardt F. Hoyer P. O. (2011). Noisy-or models with latent confounding, in Proceedings of the 27th Conference on Uncertainty in Artificial Intelligence (Corvallis, OR: AUAI Press).

[B24] Hyttinen A. Eberhardt F. Hoyer P. O. (2012). Learning linear cyclic causal models with latent variables. J. Mach. Learn. Res. 13, 3387–3439.

[B25] Kalish M. L. Griffiths T. L. Lewandowsky S. (2007). Iterated learning: intergenerational knowledge transmission reveals inductive biases. Psychon. Bull. Rev. 14, 288–294. 10.3758/BF03194066 17694915

[B26] Kim J. H. Pearl J. (1983). A computational model for causal and diagnostic reasoning in inference systems, in Proceedings of the 8th International Joint Conference on Artificial Intelligence (San Francisco, CA: Morgan Kaufmann).

[B27] Lober K. Shanks D. R. (2000). Is causal induction based on causal power? critique of cheng (1997). Psychol. Rev. 107, 195–212. 10.1037/0033-295X.107.1.195 10687407

[B28] Lu H. Yuille A. L. Liljeholm M. Cheng P. W. Holyoak K. J. (2008). Bayesian generic priors for causal learning. Psychol. Rev. 115, 955–984. 10.1037/a0013256 18954210

[B29] Lucas C. G. Bridgers S. Griffiths T. L. Gopnik A. (2014). When children are better (or at least more open-minded) learners than adults: developmental differences in learning the forms of causal relationships. Cognition 131, 284–299. 10.1016/j.cognition.2013.12.010 24566007

[B30] Lucas C. G. Griffiths T. L. (2010). Learning the form of causal relationships using hierarchical Bayesian models. Cogn. Sci. 34, 113–147. 10.1111/j.1551-6709.2009.01058.x 21564208

[B31] Lucas P. J. F. (2005). Bayesian network modeling through qualitative patterns. Artif. Intell. 163, 233–263. 10.1016/j.artint.2004.10.011

[B32] Machamer P. Darden L. Craver C. F. (2000). Thinking about mechanisms. Philos. Sci. 67, 1–25. 10.1086/392759

[B33] Martin C. B. (2008). The Mind in Nature. Oxford: Oxford University Press.

[B34] McDaniel M. A. Busemeyer J. R. (2005). The conceptual basis of function learning and extrapolation: comparison of rule-based and associative-based models. Psychon. Bull. Rev. 12, 24–42. 10.3758/BF03196347 15948282

[B35] Pearl J. (1988). Probabilistic Reasoning in Intelligent Systems: Networks of Plausible Inference. San Francisco, CA: Morgan Kaufmann Publishers.

[B36] Rescorla R. A. Wagner A. R. (1972). A theory of Pavlovian conditioning: variations in the effectiveness of reinforcement and non-reinforcement, in Classical Conditioning II: Current Research and Theory, eds Black A. H. Prokasy W. F. (NewYork, NY: Appleton-Century-Crofts), 64–99.

[B37] Shanks D. R. (1995). Is human learning rational? Q. J. Exp. Psychol. 48A, 257–279. 10.1080/14640749508401390 7610267

[B38] Sloman S. A. (2005). Causal Models: How People Think About the World and Its Alternatives. Oxford: Oxford University Press.

[B39] Spirtes P. Glymour C. Scheines R. (2000). Causation, Prediction, and Search, 2nd Edn. Cambridge, MA: The MIT Press.

[B40] Srinivas S. (1993). A generalization of the Noisy-OR model, in Proceedings of the 9th Annual Conference on Uncertainty in Artificial Intelligence (San Francisco, CA: Morgan Kaufmann), 208–215.

[B41] Steyvers M. Tenenbaum J. B. Wagenmakers E.-J. Blum B. (2003). Inferring causal networks from observations and interventions. Cogn. Sci. 27, 453–489. 10.1207/s15516709cog2703_6

[B42] Van Hamme L. J. Wasserman E. A. (1994). Cue competition in causality judgments: the role of non-presentation of compound stimulus elements. Learn. Motiv. 25, 127–151. 10.1006/lmot.1994.1008

[B43] Woodward J. (2012). Causation: interactions between philosophical theories and psychological research. Philos. Sci. 79, 961–972. 10.1086/667850

